# “The Rodney Dangerfield of Stylistic Devices”: End-to-End Detection and Extraction of Vossian Antonomasia Using Neural Networks

**DOI:** 10.3389/frai.2022.868249

**Published:** 2022-06-09

**Authors:** Michel Schwab, Robert Jäschke, Frank Fischer

**Affiliations:** ^1^School of Library and Information Science, Humboldt-Universität zu Berlin, Berlin, Germany; ^2^L3S Research Center, Hanover, Germany; ^3^Department of Philosophy and Humanities, Freie Universität Berlin, Berlin, Germany

**Keywords:** metaphor, Vossian Antonomasia, neural network, BERT, sequence tagging, binary classification, metonymy, information extraction

## Abstract

Vossian Antonomasia (VA) is a well-known stylistic device based on attributing a certain property to a person by relating them to another person who is famous for this property. Although the morphological and semantic characteristics of this phenomenon have long been the subject of linguistic research, little is known about its distribution. In this paper, we describe end-to-end approaches for detecting and extracting VA expressions from large news corpora in order to study VA more broadly. We present two types of approaches: binary sentence classifiers that detect whether or not a sentence contains a VA expression, and sequence tagging of all parts of a VA on the word level, enabling their extraction. All models are based on neural networks and outperform previous approaches, best results are obtained with a fine-tuned BERT model. Furthermore, we study the impact of training data size and class imbalance by adding negative (and possibly noisy) instances to the training data. We also evaluate the models' performance on out-of-corpus and real-world data and analyze the ability of the sequence tagging model to generalize in terms of new entity types and syntactic patterns.

## 1. Introduction

Stylistic devices are used to add meaning, ideas or emotion to what is literal or written to make it stand out. They are figurative and open a space for interpretation; their understanding often requires significant contextual knowledge. The more figurative they are, the more difficult they are for a machine to extract. In this paper, we describe different approaches using neural networks for automatic extraction of an otherwise well-studied phenomenon: Vossian Antonomasia.

In general, antonomasia is closely related to the family of metonymy. It replaces a name of a person by a phrase describing this person, for example, “the boy who lived” for Harry Potter since he survived a murder attempt on his life. *Vossian Antonomasia* (VA), also called “metaphorical antonomasia” (Holmqvist and Płuciennik, 2010), is a sub-phenomenon of general antonomasia. In contrast to a general antonomasia, a typical VA expression consists of three parts, namely *target, source*, and *modifier*. The source is a named entity, typically famous or well-known to the reader, that embodies a set of properties or characteristics used to describe another entity, the target. A context (modifier) is provided to help embedding the source's characteristics in the target's environment. For example, when Miles Davis is described as “the Picasso of jazz”, one or multiple characteristics of Pablo Picasso are invoked to describe Miles Davis. According to the article from which we took the quote, the latter is as “cool, endlessly inspired and prolific” (Kahn, 1999) as the former. The modifier “jazz” transfers these attributes into the target's context. Most of the time, however, the transferred characteristic is not mentioned explicitly and interpretation is left to the reader^1^. Unlike source and modifier, the target is not an essential part of the phenomenon. It can be left out, unknown or hypothetical, as can be seen in the following Example (1) (with **target**, source, and *modifier* emphasized):

(1) Another question is, Who will be the Walt Disney of *this era*? (NYT 2003/02/10/1463847)

Three more examples of VA show its diversity in syntax and usage and will be referred to in the sequel:

(2) If **Jones** was the Michael Jordan of *her time*, scoring at will, **Cain**, 33, is the Magic Johnson of *hers*. (NYT 1994/07/05/0697585)
(3) “**I**'m like David fighting the Goliath of *the beauty industry*,” she said in a recent interview. (NYT 1988/01/05/758388)
(4) As far as selling sneakers is concerned, Cleveland guard **LeBRON JAMES** is the *new* Michael Jordan. (NYT 2006/11/19/1806049)

The task to automatically identify and understand VA is challenging because their syntactic pattern can be ambiguous. When we read about “the Michelangelo of the Sistine Chapel”, it is apparent that in this case the Sistine Chapel cannot be a modifier, but that the expression as a whole refers to a specific period in Michelangelo's life as an artist, while “the Michelangelo of Manhattan” (NYT 1998/09/25/1049076)^2^ is meant metaphorically, a VA expression that in this case refers to a plastic surgeon (Michael Lerner) from New York. The author did not explain his intentions using this VA explicitly, but knowing Michelangelo as one of the most virtuoso artists, it is most likely that this characteristic is meant to describe Michael Lerner. Another example that demonstrates the difficulty of identifying VA, is “the George W. Bush of 2016”. Without knowing more context, it could refer to the person George W. Bush in the year 2016. Another possibility could be that this is a VA expression attributing another politician, for example, Hillary Clinton^3^, where the author compares Bush's dominating primary campaign of 2000 to Clinton's campaign in 2016. Furthermore, names of (even) famous persons are often used equivocal and thus, in some cases, the person is not even meant. Consider, for example, the phrase “the Madonna of Birth” in which “Madonna” does not stand for the singer, but the phrase is the title of a painting of Mary, mother of Jesus. Similarly, “a Napoleon of crisp pastry” is not an allusion to the French emperor, but the description of a dessert. These examples give a good impression of how rule-based approaches can easily fail.

The syntax of VA also varies, see Examples (2) to (4), which is one of the reasons why it is hard to detect automatically. Combined with the need for background knowledge to differentiate between VA and non-VA phrases, it is a non-trivial task. Its detection can help to improve other NLP tasks, such as fact extraction, machine translation, or entity disambiguation/co-reference resolution (Schwab et al., 2019). It can also provide new interesting question answering tasks or support creative natural language generation, especially in news and blog articles to generate fruitful content. Identification and extraction of VA can also be a step toward the association of properties and characteristics to entities in text.

The main contributions of this work are novel machine learning models for automatic detection and extraction of VA from texts. Two models are binary classifiers that outperform state-of-the-art approaches. The other two are sequence tagging models which detect all three parts of the phenomenon on the word level. This task is completely new and has not been studied before. In addition, we analyze the impact of training data and conduct two robustness studies. One shows the performance on out-of-corpus data and the other one shows the ability to generalize to new types of VA. A second contribution is a fully annotated dataset on the word level which emerged from the dataset in Schwab et al. (2019). The dataset is explained in detail in Section 3.2.

## 2. Related Work

Automatic detection of VA has rarely been studied. Especially the use of neural networks has not been explored in depth even though they have shown remarkable results in similar tasks, for example, metaphor detection as shown below.

A first approach to identify VA semi-automatically was presented by Jäschke et al. (2017) for German and English newspaper corpora. They applied POS tagging and NER at sentence level and identified candidates using a set of complex pre-defined patterns. Working on the New York Times corpus (Sandhaus, 2008), their method could identify 10,744 candidates of which less than 500 were actual true candidates as confirmed by human annotators. This results in a precision of less than 5%. One reason for the low precision was the matching of common phrases like “Rudolph Giuliani, the mayor of New York”.

A second semi-automatic approach was proposed by Fischer and Jäschke (2019), who focused on the pattern “the ENTITY of”. As a first step, they used regular expressions to extract only the candidate sentences with the mentioned pattern. After using Wikidata for distant supervision linking all candidates to Wikidata “human” entities, they manually created a list to exclude common false positive candidates like “the House of” or “the Prince of”, as those names matched Wikidata entities. Schwab et al. (2019) presented the first fully automated approach by extending the idea of Fischer and Jäschke (2019). They introduced three different methods. In the first approach, they extended and automated the approach from Fischer and Jäschke (2019) by introducing a popularity measure to replace the manually curated blacklist. In the second approach, they used a named entity recognition tool instead of distant supervision to identify candidates. In the third approach, they developed a binary classifier for candidate sentences using a bi-directional LSTM and non-contextual word-embeddings. The BLSTM performed best, beating the other approaches by 0.08 and 0.1 points in *F*_1_ score on the test dataset, while they did not evaluate the network's performance on out-of-corpus data. They also created a partly annotated corpus including 3,023 positive and 3,049 negative instances.

Other stylistic devices, similar to VA, have been covered extensively, for example, metaphors. Particularly, considering metaphor extraction as a sequence tagging task using neural network approaches has been studied recently. While Gao et al. (2018), Dankers et al. (2019); and Torres Rivera et al. (2020) use contextualized word-embeddings and bi-directional LSTMs in their model architecture, Dankers et al. (2019), Chen et al. (2020), Gong et al. (2020), and Liu et al. (2020) make use of pre-trained contextual language models, for instance, BERT (Devlin et al., 2019), RoBERTa (Liu et al., 2019), or XLNet (Yang et al., 2019). Metonymy resolution has also found recent attention in the NLP community with the advent of pre-trained transformer models. Employing BERT, Li et al. (2020) and Su et al. (2020) classified specific words in sentences, whereas Mathews and Strube (2021) transformed the problem into a sequence tagging task to tag each word in a sentence.

## 3. Datasets and Annotation

In the following, we present the datasets we used, our annotation scheme and how we handle special cases like incomplete VA or multiple VA within one sentence.

### 3.1. Annotation Process

The annotation process is based on the IOB tagging scheme (Ramshaw and Marcus, 1995), which is widely used for sequence tagging in NLP. The tags B-{TRG, SRC, MOD} stand for the beginning of a chunk, the tags I-{TRG, SRC, MOD} for the inside of a chunk. All other words (that do not belong to one of the VA chunks) are tagged O. The following example shows an annotated VA with the tags above the words:


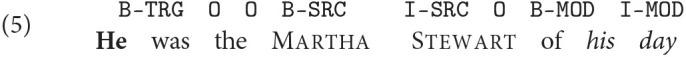


Next, we describe the annotation of the target (TRG) and the modifier (MOD), as the source (SRC) has already been identified and annotated by Schwab et al. (2019). If multiple VA appear inside one sentence, we annotate all chunks independently.

**Modifier:** The modifier is essential for VA and it always appears together with the source. As the dataset consists of specific syntactic VA patterns, the modifier's position is fixed. That is, it always appears directly after the source phrase (“the/a/an source of/for/among *modifier*”).

**Target:** Annotating the target's full name inside the article is unfeasible and not helpful for our task, since we use sentences as input sequences for the models. Instead, we annotated the chunk inside the sentence to which the source refers to directly as target. This can be the full target name itself but also references like personal pronouns (e.g., “she”, “his”), name parts (e.g., “Mr. Obama”), or descriptions (e.g., “the young student”). Consider Example (3) where the full target name of the VA is “Audrey Butvay”, but the name does not appear inside the sentence. Instead, a reference of the target name exists, namely a personal pronoun, “I”, which we annotated as target. In contrast to the source and modifier, the target appearance is optional, it does not have to appear within the same sentence or it can be missing altogether as we have explained before. After tagging, co-reference resolution could be used to identify the full target name. We did not focus on that, since co-reference resolution has already been studied deeply, see, for instance, Ng and Cardie (2002), Lee et al. (2017), or Joshi et al. (2019).

### 3.2. Annotated VA (aVA) Dataset

This is an extension of the dataset from Schwab et al. (2019) which was the result of a semi-automated VA extraction method on The New York Times Annotated Corpus (Sandhaus, 2008) which contains around 1.8 million articles that were published between 1987 and 2007. The dataset creation is based on a syntax-based approach focusing on nine syntactic patterns around the source (“the/a/an source of/for/among”, cf. Table 1 in Schwab et al., 2019). That means, every combination of the words {the, a, an} and {of, for, among} can appear around the source entity.

**Table 1:** The most frequent appearances for each VA chunk together with their frequency.

**Target**		**Source**		**Modifier**	
463 (15.1%)	He	73 (2.4%)	Michael Jordan	56 (1.8%)	His day
179 (5.8%)	-	61 (2.0%)	Rodney Dangerfield	35 (1.1%)	His time
179 (5.8%)	Him	40 (1.3%)	Johnny Appleseed	32 (1.0%)	Japan
116 (3.8%)	She	38 (1.2%)	Babe Ruth	21 (0.7%)	The 90's
107 (3.5%)	I	33 (1.1%)	Elvis	20 (0.7%)	Our time
48 (1.6%)	Her	27 (0.9%)	Mona Lisa	17 (0.6%)	China
41 (1.3%)	It	25 (0.8%)	Michelangelo	16 (0.5%)	Baseball
28 (0.9%)	You	24 (0.8%)	Cinderella	16 (0.5%)	His generation
13 (0.4%)	Me	24 (0.8%)	Madonna	16 (0.5%)	Tennis
12 (0.4%)	The man	23 (0.8%)	Bill Gates	14 (0.5%)	Her time

*The “-” in the target column indicates that there exists no target chunk in the sentence*.

We refer to the combination of the boundary words around the source as “source phrase” in the following. In a second step, the approach used Wikidata for entity linking. Here, the authors focused on entities with the property “instance of” “human”. At the end, a blacklist was manually generated to remove false positive candidates. Thus, all instances contain one of nine syntactical patterns around the source and all VA sources are linked to human entities in Wikidata. 96.3% of the instances in the dataset contain the boundary word “of” after the source. In 68.0% of those instances, there exists a VA expression. The boundary word “for” was found in 3.2% of all instances of which 6.0% contained a VA expression and “among” was found only in 0.5% of the instances where 41.0% contained a VA expression. This shows that the pattern distribution is highly unequal and the boundary word “of” dominates.

We double-checked all labels and, as described in Section 3.1, we annotated target and modifier for the positively labeled instances as the source was already annotated. Two trained^4^ annotators annotated the dataset with an inter-annotator agreement of 0.88 by Cohen's kappa. Disagreements were discussed and re-annotated by annotators and expert. During the annotation process we found and updated incorrectly labeled instances in the dataset. That included positive labeled instances that did not contain any VA expression, only syntactical patterns that looked like VA expressions. It also included falsely tagged VA expressions that appeared not to be a VA expression. Those instances were re-labeled and re-annotated. We also removed duplicates that showed up because instances appeared in multiple articles of the New York Times and therefore were intentionally not removed before. Hence, the numbers slightly differ from Schwab et al. (2019): Our updated and fully annotated dataset consists of 5,995 sentences, 3,066 of them include a VA, 2,929 do not.

Analyzing the frequency and distribution of the VA chunks in the dataset, we can observe a large diversity by dividing the number of distinct chunks by the number of all sentences containing VA: Among the 3,066 sentences that contain VA,

- 58% of the target chunks, 44% of the source chunks, 71% of the modifier chunks,
- 83% of the target-source pairs, 96% of the target-modifier pairs, 95% of the source-modifier pairs, and
- 98% of the chunk triples,

are distinct. The modifier chunk is the most diverse chunk since it is not limited to entities or pronouns but can include temporal (“the 90's”, “his era”) or local (“Europe”, “New York”, “the East”) phrases or refer to different genres (“sports”, “music”, “politics”). The source chunk has the least diversity. As one would expect, some entities are mentioned more often than others, since they stand like no other for a certain property or characteristic, for example, Michael Jordan for success. Targets are not as diverse as modifiers, since they often consist of pronouns. If we could identify the names of the referred entities, the target chunk would be even more diverse. Table 1 shows the top 10 phrases for each chunk.

### 3.3. Enriched VA (eVA) Datasets

When we analyzed the origin of the aVA dataset from Schwab et al. (2019), we discovered two biases. One bias is the limited variation of sentence structures. This is a result of the rule-based approach that was used to create the dataset. In particular, the candidates were chosen by nine syntactic patterns as explained in the previous section. Thus, all instances of the dataset, positive and negative, match one of these patterns. Second, the aVA dataset has an almost balanced ratio between positive and negative instances. This ratio does not represent the true distribution of VA in real texts. To our knowledge, the distribution of VA has not been studied on large corpora, but Schwab et al. (2019) found around 5 VA expressions per 100,000 sentences. As they focused on human entities and used the mentioned source phrases, this is a lower bound. These types of biases are common and one of the reasons why models are overfitting and therefore perform worse on data outside the corpus.

To generate a dataset that better reflects the real-world distribution of VA, we have two options. Either we annotate a very large number of random sentences, which is unfeasible, or we use one of the phenomenon's properties: The share of articles that contain VA expressions is reasonably high but the share of VA expressions per sentence is low.

In particular, we add randomly chosen sentences from the New York Times Annotated Corpus (Sandhaus, 2008) to the aVA training (75%) and test (25%) dataset, respectively, as negative instances, ensuring that none of them are already part of the aVA dataset. This method enriches the datasets in two ways: It diversifies the sentence structures of negative instances and creates an imbalance between the number of positive and negative instances. As a bonus, we get a larger training dataset and a better estimation of the model performance on new data by evaluating not only on the aVA test dataset, but on the generated test datasets. The sentences we add to the aVA training and test dataset vary from 50,000 (eVA-{TR, TE}-50) to 500,000 in steps of 50,000 sentences, reducing the fraction of positive instances from 51.1% (eVA-{TR, TE}-0) to 0.9% and 0.3%, respectively. We are aware that these datasets can be noisy, especially when we choose larger amounts of sentences but as all instances from the aVA dataset are excluded and because of the sparseness, we concluded that this is a reasonable construction of negative instances.

### 3.4. Signal (SIG) Dataset

The Signal 1 Million News Articles Dataset (Corney et al., 2016) contains around one million blog and news articles, mainly written in English and published between September 1st and 30th, 2015. As these articles are from another time period, from different countries, and from different sources, the dataset differs substantially from the NYT dataset. Thus, it is a suitable dataset for a robustness study. As the whole dataset would be too large, we extracted a random sample from the dataset. In particular, we decompressed the gzip-compressed JSONL file and read the json records line by line. From each record, we extracted the fields “content” and “title” using the NLTK sentence tokenizer (Bird et al., 2009) and took a random sample of one million sentences out of all tokenized sentences.

## 4. Methods

We first formally define the tasks we are studying and then explain our models for both tasks. All models are based on neural networks. For each task, we develop two models, one is trained from scratch and the other is a fine-tuned model that is based on a pre-trained transformer model.

### 4.1. Tasks

We aim at a fully automated extraction of VA from texts. More specifically, given a sentence *S* of words *w*_1_ *w*_2_…*w*_*n*_, we aim to solve:

**Task 1: Binary Classification:** Predict a binary label *l* indicating whether *S* contains at least one appearance of a VA or not.

**Task 2: Sequence Tagging:** Predict for each word *w*_*i*_ of *S* a tag *t*_*i*_ which indicates whether *w*_*i*_ belongs to a target, a source, or a modifier chunk or whether it is not a VA part at all.

Both tasks will be addressed and evaluated independently. Still, we can transform the results of the sequence tagging task to the binary classification task. In particular, we solve Task 1 implicitly while solving Task 2 by exploiting the definition of VA: VA consist of a source, a modifier, and an optional target chunk as explained in Section 1. Hence, if our sequence tagging models predict at least one source tag and at least one modifier tag inside a sentence, the sentence can be labeled positive, negative otherwise.

### 4.2. Binary Classification

#### 4.2.1. Baseline

Suitable baselines for this task that do not result in low precision and thus *F*_1_ score are rare. As seen in Schwab et al. (2019), rule-based approaches focusing on the source phrase, for example,

- a/an/the [entity in Wikidata]
- a/an/the [entity in Wikidata] among/for/of
- a/an/the [entity by NER] among/for/of

do not work as their performance is too low. This is because the syntactic patterns where VA sources appear in are used in different contexts as well, see Section 1. Even choosing famous entities only would not work. Take, for instance, the singer Prince, who is one of the most popular singers of the twentieth century and also popular in Wikidata according to different metrics. Extracting all patterns “the Prince of” would result in low precision since 537 out of 855 candidates would be sentences including the phrase “the Prince of Wales” which is a title given to the heir apparent of the British throne. Particularly, we did not find any VA expression within these candidates. Generating a baseline by labeling all instances based on the most frequent labels of the corresponding source or source-modifier pair does not work either, since the training dataset is too diverse (cf. Section 3)—most sources or source-modifier pairs only appear once in the dataset. Thus, we use the best performing approach from Schwab et al. (2019) as a baseline which was a neural network approach, where a bi-directional long short-term memory network was trained for binary sentence classification. This approach outperformed the rule-based approach in Schwab et al. (2019) by 0.08 points in *F*_1_ score.

#### 4.2.2. BLSTM-ATT

**Figure 1:**
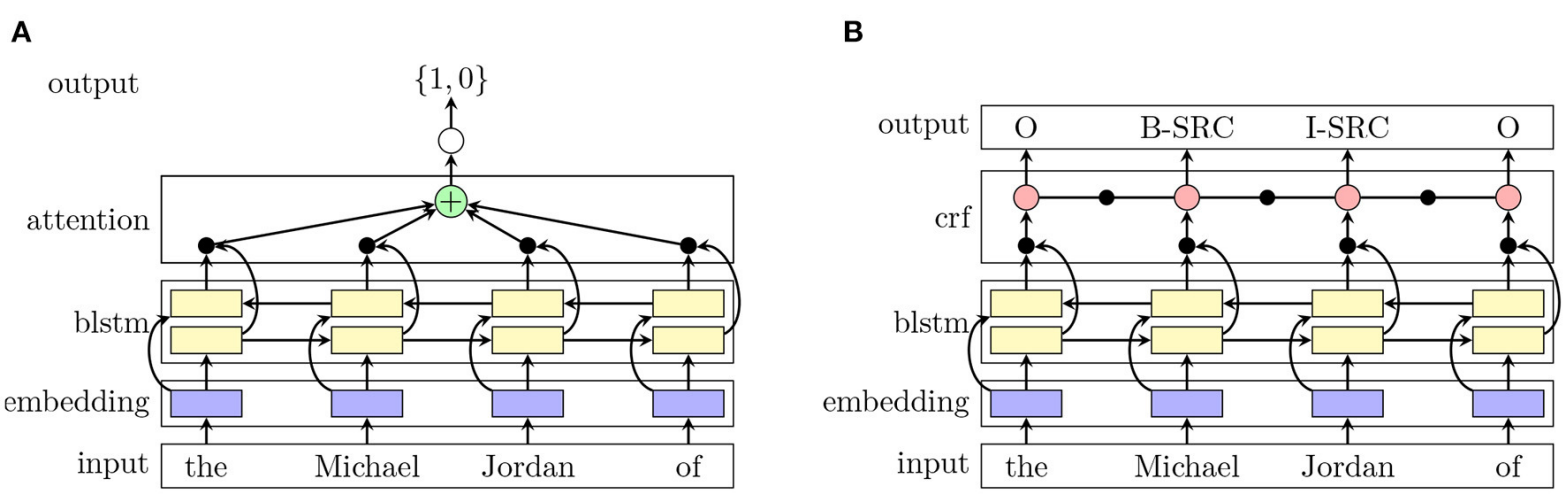
**(A)** Shows the BLSTM-ATT architecture for the classification task and panel. **(B)** Shows the BLSTM-CRF architecture for the sequence tagging task.

Long short-term memory networks (LSTM, Hochreiter and Schmidhuber, 1997) have been used widely for sequence classification tasks due to their ability to capture long-term dependencies. Their limitation of only representing a word by either its left or right context can be met by adding a second LSTM layer that reads the sequence in reverse. Subsequently, we can represent the word using both, its right and left context, by concatenating them. We extend the recurrent network architecture from Schwab et al. (2019) in two ways, similar to Gao et al. (2018). First, we expand the word representations. As in Schwab et al. (2019) we use GloVe embeddings (Pennington et al., 2014) which are non-contextualized. Peters et al. (2018) showed that contextualized word representations can improve the performance in many NLP tasks, including metaphor detection (Gao et al., 2018). Thus, we also use ELMo embeddings from Peters et al. (2018) which are deep contextualized word representations. Both embeddings are concatenated and passed to the BLSTM layers. On top, we add an attention layer that consists of a linear layer and softmax normalization (cf. Figure 1A). To compute the label of the sentence, we feed the resulting vector from the attention layer to a feedforward layer.

#### 4.2.3. BERT-CLF

This approach uses state of the art pre-trained language representations to classify sentences. Google's BERT [Bidirectional Encoder Representations from Transformers, Devlin et al. (2019)] has outperformed previous methods on a large number of NLP tasks, including sequence classification. As an unsupervised general-purpose method of pre-trained language representations, it is trained on large text corpora and can be fine-tuned for specific down-stream tasks. BERT emerged from similar contextualized language representations like ELMo (Peters et al., 2018) or ULMFit (Howard and Ruder, 2018) but is significantly stronger since it is deeply bi-directional, that is, it combines contextual information from both sides, left and right. We do not train the whole model but fine-tune the pre-trained BERT model to our task by adding a single new layer which is then trained with our labeled data.

### 4.3. Sequence Tagging

#### 4.3.1. Baseline

This task has not been studied at all, so we do not have a baseline to compare our models with. Conditional random fields (CRF, Lafferty et al., 2001) have been shown to perform well on sequence tagging tasks, such as part of speech tagging, shallow parsing or named entity recognition, see (Lafferty et al., 2001; McCallum and Li, 2003; Sha and Pereira, 2003). Hence, we train a CRF with our annotated data and use it as baseline.

#### 4.3.2. BLSTM-CRF

BLSTMs have shown remarkable results in sequence classification but also in sequence tagging tasks, since they can represent long-term dependencies as explained in Section 4.2. CRFs on the other hand are able to model the labels jointly, not independently. With this advantage, CRFs include dependencies across labels, which a standalone LSTM is not able to do. Our labels have those dependencies, for example, I-SRC can not follow B-MOD.

As shown by Huang et al. (2015) and Lample et al. (2016), a combination of BLSTMs and CRFs have improved many different sequence tagging tasks using the right and left contextual information from the BLSTM and the sentence-level tag information from the CRF. In our approach, the neural network architecture employs a BLSTM and a CRF, following Huang et al. (2015) and Lample et al. (2016). As in Section 4.2.2, we use a concatenation of GloVe (Pennington et al., 2014) and ELMo (Peters et al., 2018) embeddings to represent each word in the sentence and pass those representations to the LSTM layers, which compute two vectors as explained before. Subsequently, both vectors are concatenated and then fed to the CRF as features (cf. Figure 1B). The CRF jointly computes the tags for each word in the sequence.

#### 4.3.3. BERT-SEQ

As in Section 4.2, we use the pre-trained BERT language model (BERT-base-cased) and fine-tune it on our dataset. The top layer differs from the one in the classification task and tags every word of an input sequence instead of labeling the sentence.

### 4.4. Experimental Setup

We describe the setup of the algorithms for our experiments.

**Baseline:** We train a linear-chain CRF using the L-BFGS algorithm (Nocedal, 1980). We use fixed parameters, namely *c*_1_ = 0.1 and *c*_2_ = 0.1 for *l*_1_ and *l*_2_ regularization.

**BLSTM-ATT:** Each word is represented by a concatenation of 300d GloVe vectors and 1024d ELMo vectors. The hidden state of the bi-directional LSTM is set to 300 and dropout is applied on the inputs before the LSTM layer and after the attention layer. SGD is used to optimize the model.

**BLSTM-CRF:** We use the open source FLAIR framework (Akbik et al., 2019) and a concatenation of 300d GloVe vectors and 1024d ELMo vectors for word representations. The bi-directional LSTM hidden state is 300.

**BERT-CLF/SEQ:** We use the open source HuggingFace transformers framework (Wolf et al., 2020) and employ the pre-trained cased BERT-Base model from Devlin et al. (2019). It contains *L* = 12 transformer blocks, *A* = 12 attention heads, its hidden size is *d*_*h*_ = 768 and it has around 110*M* parameters. The model uses the Adam optimizer.

The hyperparameters for all three neural models were optimized on epoch, batch size, and learning rate on a validation set which is suggested for BERT by Devlin et al. (2019).

## 5. Evaluation and Results

Determining the recall on the whole NYT dataset is unrealistic, since it contains more than 1.8 million articles that cannot be annotated on a sentence level or even word level. Schwab et al. (2019) showed that labeling a subset does not help either due to sparseness: They labeled 105 randomly selected articles, 4,429 sentences in total, and only found one VA expression. Another attempt to reduce the number of candidate sentences to find a suitable subset for evaluating recall could be by making use of the VA definition: VA consists of at least a source and a modifier. For that reason, we excluded all sentences that did not contain a named entity or did not contain at least two words. The result was a reduction by only 25%, which did not help. Finally, we concluded that it is unfeasible for us to determine recall on a larger dataset without introducing bias.

**Figure 2:**
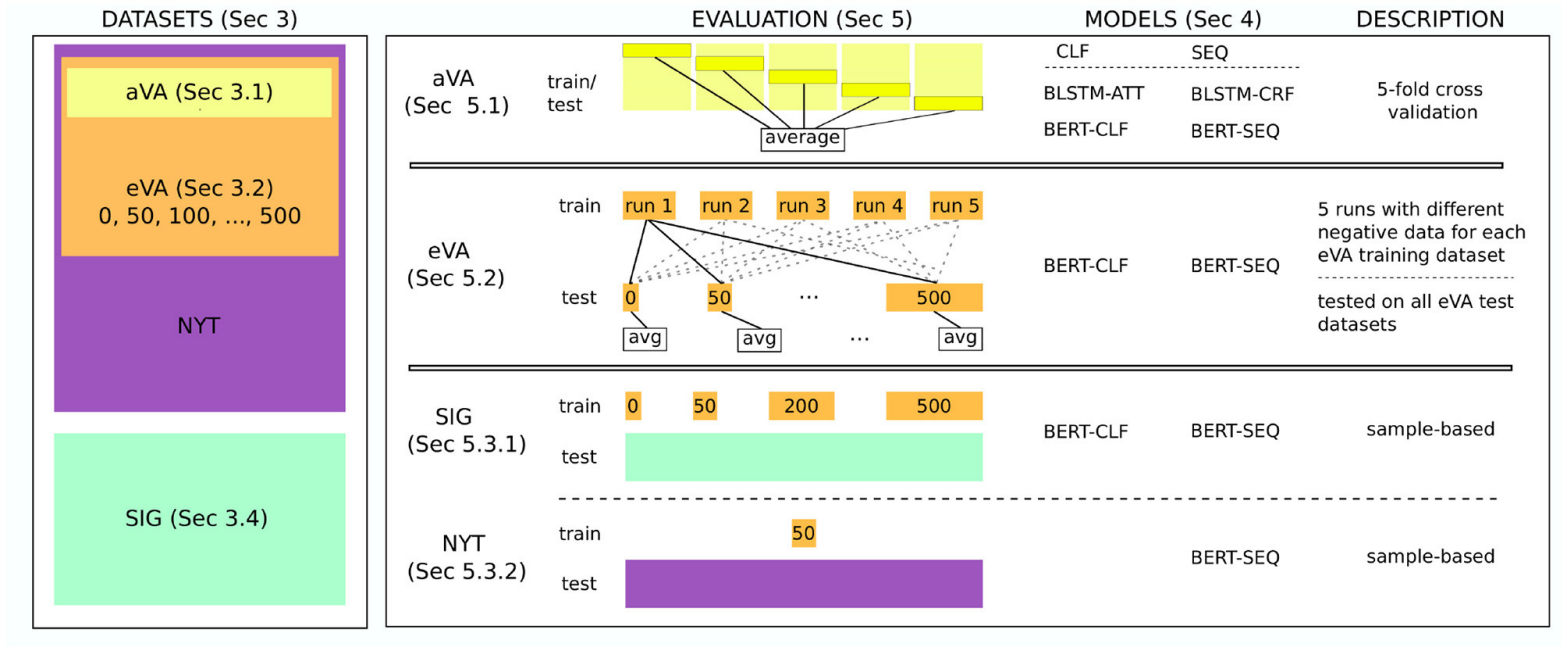
An overview of the models and datasets in the different evaluation setups. On the left side, the datasets are summarized. The aVA dataset is part of the eVA datasets which themselves emerged from the NYT dataset. The SIG dataset on the other hand is independent. On the right side, the evaluation and the models that are evaluated on the corresponding datasets are shown. For example, all models are evaluated on the aVA dataset (top row) whereas only the BERT-SEQ model was used for the robustness study on the NYT dataset (bottom row). The description summarizes how the evaluation has been conducted. All evaluations are conducted independently.

Instead, we conduct three different evaluations. First, we train and test our models on the aVA dataset using five-fold cross validation (Section 5.1). Then, we use the eVA datasets for training and testing to analyze the models' performance on datasets whose class distributions are closer to real-world data (Section 5.2). In particular, we study the effect of imbalanced and noisy training data. This is tested on different imbalanced test datasets. This evaluation is an indication on how the model performs on real-world data. Finally, we conduct two different robustness studies. In the first study, we analyze the model's performance on real-world data (Section 5.3.1). The second study analyzes the sequence tagging model and its ability to generalize to new VA expressions in terms of syntax and type of entities (Section 5.3.2). For both studies, we use samples of the outcome of the model to analyze the tasks. For those tasks, we use the best performing models from the previous evaluation. An overview of the models and datasets in the different evaluation setups are shown in Figure 2.

### 5.1. Performance on the aVA Dataset

#### 5.1.1. Classification

Table 2 shows the results of the classification task on the aVA dataset using five-fold cross validation. Both approaches, BLSTM-ATT and BERT-CLF, beat the baseline in precision, recall, and *F*_1_ score. The numbers of the baseline differ from Schwab et al. (2019) because of the updated labels in the aVA dataset as described in Section 3. BERT-CLF shows an improvement in all three measures of around 0.1 points compared to the baseline. Comparing BLSTM-ATT and BERT-CLF, we can observe that recall is similar in both approaches, but precision of BERT-CLF is higher. The reason BERT-CLF performs best could be the advantage of being trained before on a large unlabeled dataset and only fine-tuned on our task. In other words, the model benefits from the pre-training and is not only dependent on the relatively small training dataset.

**Table 2:** Performance on both tasks using five-fold cross validation on aVA.

	**Approach**	**Precision**	**Recall**	***F*_**1**_**
Classification	Baseline	0.876	0.880	0.878
	BLSTM-ATT	0.921	0.974	0.947
	BERT-CLF	0.971	0.977	0.974
	BLSTM-CRF-b	0.970	0.961	0.965
	BERT-SEQ-b	0.962	0.978	0.970
Sequence tagging	Baseline	0.765 (0.386)	0.616 (0.193)	0.682 (0.257)
	BLSTM-CRF	0.908 (0.910)	0.907 (0.730)	0.907 (0.810)
	BERT-SEQ	0.908 (0.933)	0.944 (0.831)	0.926 (0.879)

*The “b” indicates the binarized results of the sequence tagging models. The strict scores are in parentheses*.

As explained in Section 4.1, the sequence tagging models implicitly solve the classification task by transforming their tagging results into binary labels. These models are marked with “-b” for “binarized”. As shown in Table 2, both binarized models outperform BLSTM-ATT and perform almost as good as BERT-CLF.

**Error Analysis**. The prediction errors of the best approach, BERT-CLF, consist of 60% false positive errors and 40% false negative errors. Common false positive errors include sentences where entities are mentioned together with a specification of the entity, for example, “But should I be the Pierre Cardin of today…?” (NYT 2002/08/18/1416592). Without more context, it is even for humans impossible to understand the meaning of the sentence. In general, it could be a VA expression but in the context of the article, it is Pierre Cardin who is speaking of today's version of himself, so “today” is no modifier but a specification. Similar, there are cases like “an Augusto Pinochet of Chile” where the country is mentioned to identify the correct person, “the Greta Garbo of ‘Grand Hotel' ” discussing the role of Greta Garbo in the movie Grand Hotel, or “the Bill Clinton of the 1992 campaign”, specifying a period of Bill Clinton when he ran for US president. False negatives include instances whose syntax is similar to that of the mentioned false positives, for instance, “the Lana Turner of the 1990's”, “the Harold Stassen of Utah”, or “a Marx for the twentieth century”. Although the words appearing after the entities in these examples are semantically very similar to those of the false positive examples (e.g., “Utah” vs. “Chile”, or “1990's” vs. “today”), they are indeed no specification but modifiers.

#### 5.1.2. Sequence Tagging

BERT-SEQ outperforms the other models in all three measures, having an *F*_1_ score of 0.926. This is an excellent result considering the complexity of the task and the small dataset. BLSTM-CRF shows a similar precision but is lower in recall, whereas the baseline cannot compete with the proposed models. The performances of the models are summarized in Table 2. The scores in parentheses indicate a strict metric that only considers a sentence to be predicted correctly if *all* tags of all words of the sentence are tagged correctly. BERT-SEQ only loses a few points in recall and *F*_1_ score, whereas BLSTM-CRF shows a bigger gap between the two metrics, especially in the recall score. The precision, on the other hand, even increases in both models which is due the fact that in the strict metric a prediction for a sentence can only be either correct or false. In the other metric, all chunks count individually, so there can be multiple false predictions in one sentence. As a result, BERT-SEQ is also better in identifying a whole VA expression inside one sentence, that is, all chunks that belong together.

As a further step, we analyze the results of the best model, BERT-SEQ, on the chunk level in Table 3. Source prediction works best with an *F*_1_ score of 0.96, followed by modifier and target prediction. The results show that the model does not only learn syntactic rules by tagging all named entities inside a syntactic pattern as source, for example, “the entity of”, as the negative instances contain the same syntactic pattern around named entities as the positive ones (cf. Section 3). For example, the phrase “the Beethoven of” appears as part of VA expressions where “Beethoven” is the source, but also in sentences without any VA expression. This indicates that the model learns a deeper semantic understanding of the phenomenon. The high precision of the model leads to the same conclusion. The performance drop between source, modifier, and target may have different causes. One reason for the high source scores could be the limited number of syntactic patterns they appear in which makes it easier to tag all words that belong to the source chunk correctly. The property of the source of being a named entity (and in the aVA dataset even a human entity) could be another possible explanation. The modifier, on the other hand, is mostly a noun phrase that appears after the source, but consists of up to 25 words in the aVA dataset which could make it harder for the model. Still, as the modifier's position is fixed, it is easier to tag the modifier than the target. The target is not essential, hence it does not exist in each VA expression, especially not in the same sentence. For the aVA dataset, around 6% of all VA sentences do not contain a target. Also, the position of the target inside a sentence does not depend on the source and the modifier. It can stand anywhere depending on the sentence structure. The last reason for the low target score is the fact that there may be multiple target references inside the sentence and the model tags the wrong reference. The number of source and modifier chunks differ, since some VA expressions contain multiple sources [cf. Example (3)].

**Table 3:** Performance of BERT-SEQ on each VA chunk using five-fold cross validation on aVA.

**Chunk**	**Precision**	**Recall**	***F*_**1**_**	**Count**
Target	0.851	0.906	0.878	2,620
Source	0.945	0.977	0.960	2,798
Modifier	0.917	0.957	0.936	2,786

**Figure 3:**
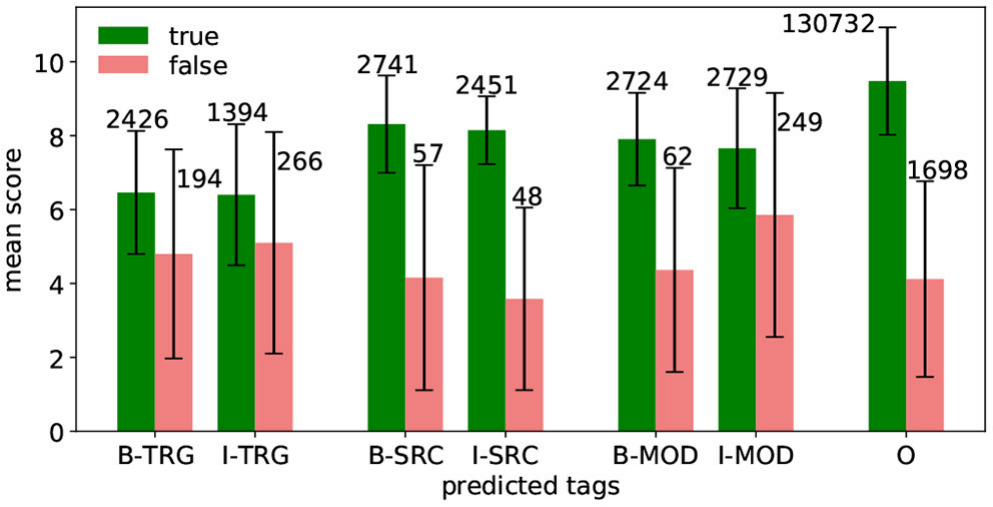
Prediction margin of predicted tags using five-fold cross validation on aVA.

For each word, the model does not only return the predicted tag but a score vector where each entry corresponds to one of the tags. The tag corresponding to the highest score in the vector is then predicted. We also measure the model's prediction margin δ_*w*_ of a predicted tag for word *w* using the minimum margin-based sampling method from Schein and Ungar (2007):


(1) $$\begin{array}{l} \delta_{w}=|\hat{P}\left(t^{\prime }\;|\;w\right)-\hat{P}\left(t^{\prime \prime }\;|\;w\right)|\text{,} \end{array}$$

where $\hat{P}\left(t\;|\;w\right)$ is the prediction score of tag *t* for word *w*, and *t*′ and *t*″ are the two most likely tags. In other words, δ_*w*_ is the distance between the highest and second highest score and we interpret it as the model's confidence. Figure 3 shows the mean and standard deviation of δ for each tag. The bar length represents the mean, the whiskers stand for the standard deviation. The numbers above the bars show the number of appearances.

In general, the model is most confident in predicting O-tags, as their mean margin score is by far the highest. Also, the figure underlines the results from Table 3, namely, the model is most confident in predicting SRC-tags, followed by MOD-tags, and TRG-tags. For all tags, it holds that the mean of the false prediction scores is lower and the standard deviation is higher compared to their true prediction counterparts. This implies that the model is less confident about false predictions. The true predicted B-tags and the corresponding I-tags have similar scores—which indicates that the model is as confident in identifying the first word of a chunk as in identifying the following words.

**Error Analysis**. For BERT-SEQ we can analyze the errors on the word level and also distinguish between errors on the different VA chunks. Table 4 summarizes the different error types for each chunk. The false positive errors are split into three types. The first type, *position*, indicates errors where a chunk appears inside a sentence but the model tagged the wrong words, for example, “He called him the Michael Jordan of China” where “him” is the target chunk but the model tagged “He” as target. The type *partly* indicates that parts of the prediction and the corresponding chunk are overlapping, but not every part of the chunk is tagged correctly, for instance, the model tagged only “Jordan” as source but the whole source chunk consists of “Michael Jordan”. Finally, the type *complete* stands for the prediction of a chunk in a sentence where the chunk does not appear at all. This happens, for example, in sentences that do not contain a VA expression but the model predicted VA chunks.

**Table 4:** Distribution of error types of BERT-SEQ in all five-folds.

**Error type**			**Target**	**Source**	**Modifier**
False positive	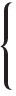	Position	41.4%	15.0%	33.6%
		Partly	13.9%	1.0%	0.4%
		Complete	27.6%	60.4%	47.4%
False negative			17.1%	23.7%	18.7%
Total errors		445	207	268	

Most source and modifier errors are of type complete and these errors appeared mostly together in the same prediction. That means, when there was a false source prediction of type complete, in more than 80% of the cases, there was also a false modifier prediction of type complete in the same sentence. In particular, for those cases, the model tagged words between one of the syntactic source phrases the training data sources appeared in as source and the words that came after the pattern as modifier. The falsely identified source words are named entities most of the time and the identified modifier is the noun phrase following the pattern, for example, “the Donald_src_ Trump_src_ of private_mod_ jets_mod_” (= a specification of Donald Trump) or “a Napoleon_src_ of eggplant_mod_” (= a kind of sandwich), or “a James_src_ Bond_src_ for the_mod_ 21st_mod_ century_mod_” (= the movie Casino Royal). Again, reading those phrases without any context could lead to the impression that they could be VA records, but in the context of the article, it is clear that they are not. Most target errors, however, are of the position type. Often, the wrong pronoun or a false named entity was tagged.

### 5.2. The Effect of (Im)balanced Training Data

In this section, we use the imbalanced eVA datasets from Section 3 to analyze the models further. By adding randomly sampled sentences as negative instances to the aVA training and test datasets, we change the datasets in two aspects: their sizes and their class distributions. In particular, for each eVA size (0, 50, …, 500) we create five training dataset versions by adding different randomly selected sentences for each version to the aVA training dataset. We use the five dataset versions to make sure that the choice of the added negative instances are not biasing the models during the training process. The datasets are named according to the size of the added instances (e.g., for eVA-TR-50 we add five times 50,000 different sentences to the aVA training dataset to create five different versions of eVA-TR-50). Then, we train the best performing models from Section 5.1, namely BERT-CLF and BERT-SEQ, on these datasets and evaluate each on all eVA-TE datasets (eVA-0-TE to eVA-500-TE). For each eVA size, we then compute the mean and standard deviation over the performances for all five runs. Subsequently, we refer to (the average performance of) these trained models as BERT-{0,50,…,500}-{CLF,SEQ} according to the size of the corresponding eVA-TR dataset.

We assume that training the models on the eVA-TR datasets will prevent overfitting and therefore result in better performance, especially on real-world data. Together with the eVA-TE datasets, we analyze the ability of the model to predict data that reflect a better real-world class distribution without additionally annotating a huge amount of data. Since we assume that all added instances are negative, the results have to be seen as an indication, but can still be very conclusive.

#### 5.2.1. Classification

**Figure 4:**
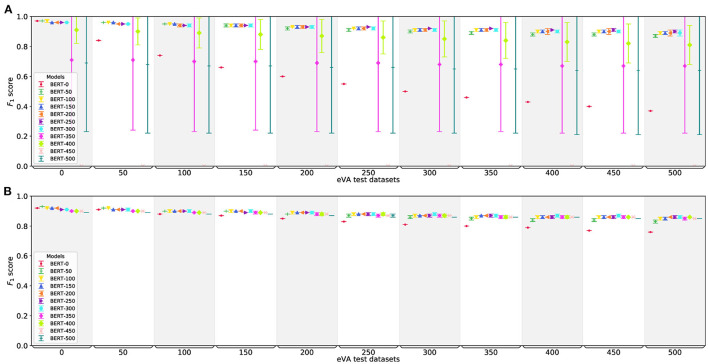
Performance of BERT-CLF **(A)** and BERT-SEQ **(B)** trained and tested on different eVA datasets. The horizontal axis shows the different eVA test datasets, the model names represent the eVA training datasets the model is fine-tuned on. All models are trained five times with five different versions of each eVA training dataset. The figure shows the mean (horizontal line) and standard deviation (whiskers) of these results.

The performance of BERT-0-CLF, our base model, falls rapidly with the increase of added negative instances in the test dataset down to 0.37 in *F*_1_ score on eVA-TE-500, see Figure 4A. This demonstrates that this model is able to show high result scores on a test dataset similar to the training data, namely the eVA-TE-0 dataset, but it is not robust on datasets that are substantially different in class distribution and sentence structure. In particular, the precision of the model is dropping which indicates that the model is overfitting. All models between BERT-50-CLF and BERT-300-CLF are more robust. While they are almost as good as BERT-0-CLF on the eVA-TE-0 dataset, they outperform BERT-0-CLF on the other eVA-TE datasets by a large margin. More specifically, the *F*_1_ score of all models decreases slowly with the size of the test datasets but stays above 0.87 on all eVA-TE datasets. This shows that the models are able to identify sentences including VA expressions, even if the class distribution is different and the negative instances are more diverse. The models are also robust against the choice of the added instances, as their standard variation is small. BERT-350-CLF and all following models perform worse. Either they have huge differences in their results depending on the version's choice of the added negative instances, as can be seen by the whiskers of the models, or they predict negative labels only, see BERT-450-CLF. The noise of the added data is one reason that could cause these results, as it is possible that there are VA records in this data which are labeled negative. This can lead to a noisy fine-tuning of the model. Another point to consider is the large class imbalance of the training dataset which can influence the classifier in a way that it predicts negative labels only.

Comparing the results to the binarized version of BERT-SEQ, BERT-SEQ-b (omitted for brevity), all models of BERT-SEQ-b equal or outperform the corresponding BERT-CLF models on almost all datasets. The most robust models are the models BERT-{300,…,400}-SEQ-b with *F*_1_ scores only decreasing from 0.96 on the eVA-TE-0 dataset to 0.91 on the eVA-TE-500 dataset, whereas the most robust BERT-CLF model is BERT-250-CLF with an *F*_1_ score of 0.95 and 0.90 on eVA-TE-0 and eVA-TE-500, respectively.

Summing up, the idea of adding randomly selected sentences as negative instances to the datasets has a huge positive effect (up to a certain point), as the models are much more robust against data that is more similar to real-world data. Another surprising result is that the implicit BERT-SEQ-b models are better classification models than BERT-CLF. As we define BERT-SEQ-b from BERT-SEQ with a loose definition of only needing a predicted source tag and a predicted modifier tag, the sequence tagger does not have to tag each word correctly to get a true binarized prediction.

#### 5.2.2. Sequence Tagging

Similar to BERT-0-CLF, the performance of BERT-0-SEQ also decreases with the increase of negative added instances in the test dataset. Figure 4B shows that all other models (BERT-50-SEQ to BERT-500-SEQ) are—in contrast to BERT-CLF—more robust. The performance of all models decreases only little with the increase of the test dataset sizes. The results on eVA-Te-500 stay between 0.73 (BERT-0-SEQ) and 0.86 (BERT-{200, 250, 300, 400}-SEQ) in *F*_1_ score. Notably, all models perform best on eVA-TE-0 although the class distribution is substantially different for most models. BERT-50-SEQ even beats BERT-0-SEQ on the eVA-TE-0 dataset. As the whiskers, and hence the standard deviation of all five runs for all models, are small, the choice of the added instances does not have any effects on the results.

In general, BERT-SEQ seems to handle class imbalance better than BERT-CLF in terms of the decrease of the result scores. One possible reason could be that the sequence tagger processes more information in the learning phase, whereas the classifier averages the scores of all words of a sentence. The sequence tagger, on the other hand, uses each word information individually. The other reason could be the effect of hyperparameters. We used the hyperparameters we got from Section 5.1, since it was unfeasible to fine-tune hyperparameters on each dataset for each model. Both aspects could lead to a worse performance of the classifier.

Putting all together, the present study confirmed our assumptions. Adding random negative instances to the training dataset is leading to a small decrease in the performance on eVA-TE-0 but has a huge robustness effect in both tasks for predicting unseen and more diverse negative instances without loosing the ability to identify positive instances.

### 5.3. Robustness Studies

In the following, we conduct two different robustness studies. The first study is an analysis whether the models are robust against new data. The second study focuses on the ability of the sequence tagging model to find new VA in terms of new source patterns and new sources, as this shows the generalization ability of the model.

#### 5.3.1. Robustness Study on Out-of-Corpus Data

We conduct a robustness study to analyze the models' performance on out-of-corpus data. For this, we train BERT-CLF and BERT-SEQ on four of the training datasets, namely eVA-TR-0, eVA-TR-50, eVA-TR-200 and eVA-TR-500, and evaluate them on the SIG dataset which is different to the NYT dataset in many aspects as explained in Section 3. Different to the training data generation in the previous section, we use the complete aVA data for the generation of the training datasets. As the SIG dataset is neither labeled nor annotated, we cannot evaluate precision, recall, and *F*_1_ score on the complete data. Still, we have to use a relatively large dataset because of the phenomenon's sparseness. Thus, we perform a sample-based evaluation.

##### 5.3.1.1. Sample Selection

First, all trained models predict labels or tags for the whole SIG dataset (one million sentences).

The number of VA predictions are summarized in Table 5 for both tasks. As we can see, the number of predicted positive labels and tags decreases by the number of added negative training data to the model.

**Table 5:** Number of positive labels and tags that were predicted from the different models for both tasks.

	**Model**	**TRG**	**SRC**	**MOD**	**Positive label**
Classification	BERT-0-CLF	-	-	-	754
	BERT-50-CLF	-	-	-	192
	BERT-200-CLF	-	-	-	90
	BERT-500-CLF	-	-	-	88
Sequence tagging	BERT-0-SEQ	179	176	201	-
	BERT-50-SEQ	131	138	125	-
	BERT-200-SEQ	75	104	101	-
	BERT-500-SEQ	54	84	80	-

We then select samples for manual evaluation based on the predictions and the prediction scores of the models. We normalize the output scores of the models using a softmax function. The computed values are used as the prediction scores. For the classification models, we get a numerical value per class that ranges between 0 and 1 and both values add up to 1. For the sequence tagging models, we get a vector where each entry corresponds to one of the IOB tags. The value range is also between 0 and 1 and the vector entries add up to 1. We want to achieve a selection which includes instances having different labels or tags but also different prediction margins to gather confident and unconfident predictions. As the predictions are not equally distributed, a random or linear selection would not fit. Consequently, we select samples as follows.

**Figure 5:**
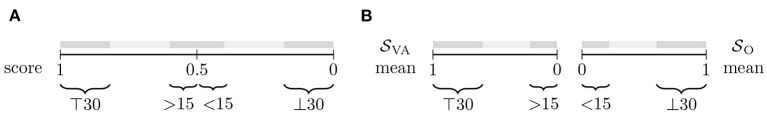
Selection of samples for evaluation of the classification task **(A)** and of the sequence tagging task **(B)**.

**Classification**. We order all predictions by their prediction score (cf. Figure 5) and then select samples based on the following two assumptions:

- The model is confident when the prediction score is close to the boundaries, 0 and 1.
- The model is not confident when the prediction score is close to the threshold, 0.5.

Thus, we choose four types of samples as follows:

- ⊤30: 30 instances whose score is highest.
- >15: 15 instances whose score is closest to but above the threshold.
- <15: 15 instances whose score is closest to but below the threshold.
- ⊥30: 30 instances whose score is lowest.

These samples cover *confident* predictions, namely ⊤30 (positive) and ⊥30 (negative), but also *unconfident* predictions close to the threshold, >15 (positive) and <15 (negative). The number of positive and negative predictions is equal.

**Sequence Tagging**. As in Section 5.1, we compute δ_*w*_*i*__ for each word *w*_*i*_ of sentence *S* = *w*_1_ *w*_2_…*w*_*n*_. Then we compute the mean μ of all δ_*w*_*i*__ for a sentence *S*:


(2) $$\begin{array}{l} \mu_{s}=\frac{1}{n}\overset{n}{\underset{i=1}{\sum }}\delta_{w_{i}}. \end{array}$$

The mean μ_*s*_ represents the model's confidence of all predicted word tags for sentence *S*. In the following, we use those measures to select samples based on the model's confidence. Before ordering the predictions, we split them into two sets. The first set $S_{\text{VA}}$ contains all sentences with at least one predicted source tag and at least one predicted modifier tag. Thus, the set consists of all instances that were (implicitly) regarded to contain VA records by the model. The split is done to make sure that we select instances containing VA tags because of the unequal distribution of predictions and the higher mean scores of O-tags. The second set $S_{\text{O}}$ contains all other sentences. For the selection of samples, we use the assumption that the model is confident about its predictions if the mean μ is high. Otherwise, if μ is low, the model is unsure. That is, if the mean is high, the δ values in the sentence are high which implies the model is confident about the choice of each predicted tag.

Then, we order both sets according to μ and select the following samples (cf. Figure 5):

- ⊤30: 30 instances whose score is highest in $S_{\text{VA}}$.
- >15: 15 instances whose score is lowest in $S_{\text{VA}}$.
- <15: 15 instances whose score is lowest in $S_{\text{O}}$.
- ⊥30: 30 instances whose score is highest in $S_{\text{O}}$.

⊤30 and ⊥30 cover the instances where the model is confident about the predicted tags and >15 and <15 contain those where it is not confident.

##### 5.3.1.2. Evaluation

Overall, we select 720 instances: 360 per task and 90 per model. Those instances are labeled and annotated by three trained annotators. They achieved an inter-annotator agreement (IAA) calculated by Fleiss' kappa of 94.1% for the classification task and 82.9% for the sequence tagging task. For the sequence tagging task, the IAA for the source was highest (84.7%), for the modifier it was lowest (79.7%), and for the target it was 84.0%. As we can see, modifier detection was most complicated for the annotators. The main reason was disagreement over the ending of the chunk. All instances that were not annotated unanimously were discussed to find consensus and then re-annotated.

**Classification**. The results of the BERT-CLF models, shown in Table 6, are fairly different on the SIG dataset compared to the eVA datasets in Section 5.2. BERT-0-CLF and BERT-500-CLF perform better than on most eVA-TE datasets, whereas the results of BERT-50-CLF and BERT-200-CLF are worse. This surprises, as the class distribution of the SIG dataset is probably even more unequal than the eVA datasets. One reason why the results are different is the fact that the models predicted correct labels for a few confident instances (⊤30) which has a huge impact in this sample-based evaluation. BERT-50-CLF achieved the best *F*_1_ score, namely 0.815, but the differences of the models are not substantial. There is a trend that recall decreases with the increase of training data while precision is increasing except for BERT-500-CLF where the precision falls as well.

**Table 6:** Performance on both tasks on the samples of the SIG dataset.

	**Model**	**Precision**	**Recall**	***F*_**1**_**
Classification	BERT-0-CLF	0.644	1.000	0.784
	BERT-50-CLF	0.733	0.917	0.815
	BERT-200-CLF	0.778	0.833	0.805
	BERT-500-CLF	0.756	0.773	0.764
	BERT-0-SEQ-b	0.689	0.912	0.785
	BERT-50-SEQ-b	0.800	0.947	0.867
	BERT-200-SEQ-b	0.756	0.919	0.829
	BERT-500-SEQ-b	0.756	0.829	0.791
Sequence tagging	BERT-0-SEQ	0.574	0.780	0.661
	BERT-50-SEQ	0.681	0.793	0.733
	BERT-200-SEQ	0.617	0.807	0.700
	BERT-500-SEQ	0.679	0.704	0.691

The binarized BERT sequence tagging models show better results, they beat all BERT-CLF counterpart models in *F*_1_ although the models were not explicitly trained for this task. BERT-50-SEQ-b performs best resulting in an *F*_1_ of 0.867.

Analyzing the accuracy of each of the samples, we see in Table 7 that the predictions in ⊥30 are all correct for all models. That was expected, as random sentences were easiest to predict as negative. Most difficult was the prediction of the samples >15 which included the sentences that were predicted positive from the model. As they were closest to the threshold, the model was most unconfident about them, and thus it is expected that they were hardest to predict.

**Table 7:** Accuracy on each sample on the SIG dataset.

	**Model**	**⊤30**	**>15**	**<15**	**⊥30**
Classification	BERT-0-CLF	0.933	0.067	1.000	1.000
	BERT-50-CLF	0.967	0.267	0.800	1.000
	BERT-200-CLF	0.967	0.400	0.533	1.000
	BERT-500-CLF	0.900	0.467	0.333	1.000
	BERT-0-SEQ-b	0.900	0.267	0.800	1.000
	BERT-50-SEQ-b	0.967	0.467	0.867	1.000
	BERT-200-SEQ-b	0.867	0.533	0.800	1.000
	BERT-500-SEQ-b	0.900	0.467	0.533	1.000
Sequence tagging	BERT-0-SEQ	0.733	0.000	0.200	1.000
	BERT-50-SEQ	0.767	0.670	0.533	1.000
	BERT-200-SEQ	0.633	0.267	0.200	1.000
	BERT-500-SEQ	0.600	0.200	0.400	1.000

Overall, the results of this section underline the results from the previous (sub-)sections, namely, that BERT-SEQ-b outperforms BERT-CLF and is more robust against real-world data. One reason could be that it is easier for the model to predict single words correctly even if not all words are predicted correctly, as it is more differentiated than predicting a label for a whole sentence.

The errors in this study tend to be false positive errors (70%) including sentences with terms like “the dark side of Europe”, “the Tiger of Asia”, or “the WINNER of Celebrity Big Brother” where the syntax is similar to the most occurring source phrases in the training data, “the source of”.

**Sequence Tagging:** As Table 6 shows, all scores are lower compared to those in Section 5.2. For example, *F*_1_ for BERT-0-SEQ drops to 0.661 from above 0.92 on eVA-TE-0. BERT-50-SEQ performs best, its *F*_1_ drops only to 0.733. As in the previous section, BERT-0-SEQ performs worst which supports our assumption that the model is overfitted and not as good as the other models for predictions on out-of-corpus data.

In Table 7, the different samples are analyzed and, as in the classification task, >15 and <15 are most difficult to predict. In contrast to the classification task, the prediction on ⊤30 seems to be more difficult which we attribute to the complexity of the sequence tagging task. Analyzing the chunks individually, Table 8 shows that in general, predicting the source is easiest, while predicting the target is most complicated. This confirms the observations from Section 5.1.

**Table 8:** *F*_1_ of all VA chunks per model.

**Model**	**Target**	**Source**	**Modifier**
BERT-0-SEQ	0.588	0.738	0.659
BERT-50-SEQ	0.659	0.773	0.764
BERT-200-SEQ	0.667	0.688	0.742
BERT-500-SEQ	0.578	0.745	0.739

Again, we analyze the errors more deeply on the word level. For all models, most errors of each chunk were false positive errors of type complete, where the model predicted the chunks in sentences not including any VA expression. The syntax of the prediction words inside those sentences are similar to many VA expressions, for example, “Velvetbandit_trg_ is our LomoHome_src_ of the_mod_ Day_mod_”, in which “LomoHome” is an award from a website, or “Alice_trg_ Cooper_trg_ is the God-voice_src_ of rock_mod_”, where “God-voice” is no entity and thus, cannot be a source of a VA expression.

#### 5.3.2. Generalization Study

In the sequence tagging task, we can differentiate between two types of VA generalizations in predictions:

1. New (unseen) syntactic VA patterns: We focus on source phrases, that is, the boundary words around the predicted source, as the modifier and target are typically not part of common patterns.
2. New (unseen) chunks: We focus on sources, as the source is the key component of any VA.

The BERT-50-SEQ model tags all sentences of the NYT dataset that have not been included in the eVA-TR-50 dataset, as this dataset is used for training the model. We use this model, as it achieved high results in the previous experiments. Since we are only interested in new and unseen VA records, and to make sure that the model receives enough sentences with possible syntactic variants of VA expressions, we use the NYT data for this study. In total, the model tagged more than 60,000,000 sentences on the word level. 9,578 sentences were tagged with at least one source tag and one modifier tag which we defined as a positive prediction. Since we cannot annotate all predicted sentences, we conduct a sample-based evaluation.

##### 5.3.2.1. Sample Selection

**New source phrases**. The training dataset consists only of VA expressions that contain one of the source phrases “a/an/the source among/for/of”. Thus, we here focus on predictions where the source is enclosed by different boundary words. Table 9 reports the top 10 predicted source phrases, split into seen and unseen phrases based on the training dataset. We can see that most predictions contain one of the seen source phrases, especially “the-of”. This was expected, as the majority of VA expressions in the training data also contain this pattern. The majority of the unseen predicted source phrases only appear once or twice. One reason is that the model does not always tag the whole source correctly. Consequently, when extracting the source phrase, that is, the words surrounding the words tagged as source, it can contain parts of the actual source. Therefore, we ensure to include frequent phrases in our sample as well, since those phrases are more reliable as correct phrases around a predicted source. Consequently, we randomly choose 25 sentences from the 10 most frequent phrases surrounding the words tagged as source that have not been in the training dataset (the “frequent” sub-sample) and 25 instances from predicted source phrases that appeared only once in the predictions (the “rare” sub-sample).

**Table 9:** Top 10 predicted source patterns [split into seen (only nine exist) in the training data and unseen (new)] and top 10 predicted VA chunks.

**Source patterns**				**Chunks**					
**Freq**	**Seen**	**Freq**	**Unseen**	**Freq**	**Target**	**Freq**	**Source**	**Freq**	**Modifier**
6,895	the - of	104	of - of	2,419	-	116	Holy Grail	322	the world
393	a - of	104	and - of	450	he	88	Cadillac	157	the
79	a - for	62	the - in	299	it	85	Pied Piper	81	the 90s
60	an - of	61	as - of	210	him	71	Rolls-Royce	63	New York
34	the - for	57	a - in	128	they	60	Paris	55	America
21	a - among	31	or - of	122	this	58	Harvard	48	this world
14	an - for	29	- of	101	i	43	Microsoft	45	our time
13	the - among	29	this - of	94	we	42	Venice	44	his day
2	an - among	26	a - on	91	she	39	Demon Barber	43	the East
		25	was - of	54	her	37	Switzerland	41	its day

**New sources**. In Table 9, the 10 most frequent tagged chunks are listed. As we can see, target and modifier chunks are overlapping with the most frequently used modifier and target chunks from the annotated dataset in Table 1, whereas the tagged source chunks are not as they do not consist of human entities. Again, we choose 25 instances that include one of the 10 most frequently tagged sources that are not already in the training data (“frequent” sub-sample) and 25 instances where the tagged source only appeared once in the predictions (“rare” sub-sample). With this selection, we can analyze the model's ability to predict new sources in general but also whether frequently predicted sources are more likely to be part of correctly predicted VA compared to rare sources that are predicted just once.

##### 5.3.2.2. Evaluation

Annotating both samples by the same annotators as in the previous study, we get an IAA by Fleiss' kappa of 0.78. The score is lower than before, since there was a misunderstanding in the annotation process which affected target and modifier annotation. In particular, the article before the noun of the target and modifier was not tagged by one of the annotators. Taking this error out, we get a kappa score of 0.84 which is similar to the agreement in the previous robustness study. All disagreements were discussed and re-annotated.

**New source phrases**. The model achieves an *F*_1_ score of 0.574 (0.75 binarized), see Table 10. Compared to the evaluations before, the scores are substantially lower. This indicates that it is more difficult to predict VA expressions having different syntactic patterns around a predicted source compared to the training data. This is expected, as the model has never seen these types of VA in the training data. Comparing the score with the binarized score, there is a huge gap and the binarized score is much higher. Consequently, we can conclude that the model often did not tag all words belonging to the chunks correctly but parts of it. Hence, it could predict at least one correct source tag and one correct modifier tag if there appeared a VA expression in the sentence. Looking at the predictions of the chunks independently, the model shows a similar behavior to the previous evaluations. Mainly, source prediction worked best, whereas target prediction was most difficult.

**Table 10:** Evaluating predictions of BERT-50-SEQ from the NYT dataset in terms of new and unseen source phrases and sources, respectively.

		**Precision**	**Recall**	***F*_**1**_**
New source phrases	Total	0.471 (0.600)	0.733 (1.000)	0.574 (0.750)
	TRG	0.385	0.652	0.484
	SRC	0.529	0.794	0.635
	MOD	0.480	0.727	0.578
New sources	Total	0.712 (0.760)	0.876 (1.000)	0.786 (0.864)
	TRG	0.667	0.743	0.703
	SRC	0.740	0.949	0.831
	MOD	0.720	0.923	0.809

*The label “total” represents the micro average. The binarized scores are in brackets*.

Out of the 25 instances from the “frequent” sub-sample, 17 consist of a VA expression. In contrast, the sub-sample with rare source phrases contains only 13 VA expressions. This indicates that frequent source phrases are more often correct. The boundary word after the predicted source also seems to have an impact. While the word “of” appeared 34 times (21 positive), “in” appeared 8 times having 4 VA expression. The word “on” only appeared two times but both times a VA expression was predicted correctly. Interestingly, nine of the thirteen rare phrases that contained a correct VA source actually consist of an extension of an already seen source phrase, for example, “the soft-spoken Barnum of the restaurant world” or “the deadly ‘Big Brother' of sports”. In these cases, a word, that in some of the cases is even part of the modifier, is inserted between the first boundary word and the source. Consequently, this inserted word was detected as part of the source phrase. The model also found completely new VA structures like “a Nepalese Robin Hood facing down corrupt and ineffective governments” where “Nepalese” is the modifier standing before the source “Robin Hood”. The model only identified the source correct and tagged “corrupt and ineffective governments” falsely as modifier. In this case, the phrase after the source is an explanation of this VA rather than a modifier. These findings suggest that the model is able to generalize to new VA types even if the whole chunks are not tagged correctly. This can be used to find syntactically new VA patterns which can be leveraged for future analyses.

Typical false positive errors included established terms like “the Nobel_src_ in economic_mod_ science_mod_” where the model falsely tagged “Nobel” as source and “economic science” as modifier or “Kitty_src_ Bethe_src_ of Manhattan_mod_”, where the model tagged “Kitty Bethe” as source and “Manhattan” as modifier but Manhattan turned out to be the residence of the woman Kitty Bethe.

**New sources**. We used two approaches to analyze the model's ability to predict unseen sources. One way is to split the aVA dataset into training (80%) and test (20%) data such that the test data does not include any source entities from the training data. The scores are similar to the five-fold cross validation scores from Section 5.1.2 with an *F*_1_ score of 0.908 which gives a first clue on the ability of the model to predict VA with unseen sources. Still, training and test data only contain human entities as sources—this could lead to an easier prediction compared to other named entity types. Hence, we conduct another sample-based evaluation to understand whether the model is able to identify source entities that are not limited to humans.

Here, the model achieves an *F*_1_ score of 0.786 (0.864 binarized) which indicates that it can successfully predict VA with unseen sources that are not humans. One reason is the fact that most correct predictions consist of an already seen source phrase (33 out of 38) which shows that the phrase around a source has a huge impact on new predictions. Being more specific, the “frequent” sub-sample only contains instances having the source pattern: “a/the source of”, which is the most frequent pattern in the training data as well. In this sample, 18 (23 binarized) out of 25 instances are predicted correctly. The “rare” sub-sample, however, only contains 9 (15 binarized) correctly predicted instances. Here, 18 instances have already seen source phrases, but there are some exceptions. On the chunk level, we can see the differences in Table 10. In particular, source prediction was easiest and target prediction hardest which matches the previous analyses.

A common false positive prediction in the “frequent” sub-sample was “Sweeney Todd, the Demon Barber of Fleet Street” referring to a musical. In the “rare” sub-sample common false positive source predictions included titles (“emperor”) or consisted of an entity with a specification, falsely tagged as source and modifier, for example, “Forest Sawyer of Arabia”. Analyzing the most frequently tagged sources, they mainly consist of brands (Cadillac, Microsoft, McDonald's), fictional characters (Pied Piper, Darth Vader), or locations (Paris, Mount Everest, Switzerland).

## 6. Discussion

We presented four new state-of-the-art models for the identification of the stylistic device Vossian Antonomasia for two different tasks: binary sentence classification and sequence tagging. First, we could boost the *F*_1_ score for the classification task to 0.974 (compared to 0.878 by Schwab et al., 2019) by fine-tuning BERT. The results in the sequence tagging task are even more remarkable. The tagger is able to identify all three VA chunks, namely target, source, and modifier, in the sentence on the word level. That allowed us to analyze the phenomenon more deeply because we could study all chunks independently. Furthermore, the identification of different parts of a VA on a large scale now enables research from other disciplines (e.g., cultural studies). Again, fine-tuning BERT achieves the best *F*_1_ score of 0.926. We also showed that the sequence tagging model is able to solve the classification task implicitly by transforming the output of this model to binary labels. This works almost as good as the classification models on the annotated corpus and even outperforms the best classification model on new data.

Another result is the positive effect of changing the class distribution and the diversity of negative instances in the training data with unlabeled and possibly noisy data which resulted in the best performance on out-of-corpus data for both tasks. The models that were trained only with the annotated data could not compete with the models trained with the noisy data on more diverse test data. The idea of using random sentences as additional negative training data is simple but promising not only for the extraction of VA but for tasks in general where the target classes are rare on the sentence or word level (which is true for many stylistic devices in linguistics, but also in other areas) and should be explored further in future work of sparse phenomenons. However, for tasks like named entity recognition it probably would not work, since named entities appear frequently on the sentence level such that the unlabeled data would also be too noisy.

The robustness study on out-of-corpus data revealed that our models are able to predict VA on different data in terms of origin (articles from all over the world), style (local news and blog articles), and publication date (more than 8 years after the training data). For both tasks, the performance on the SIG data is lower than on the annotated data. Still, some of the models were able to achieve strong results. Especially the sequence tagging model trained with a noisy dataset, BERT-50-SEQ, reached the best results for both tasks, namely a binarized *F*_1_ score of 0.867 for the classification and 0.733 for the sequence tagging task. The models trained on the annotated data exclusively, however, could not achieve these scores. The gap between the binarized and sequence tagging results shows that the model is able to identify sentences containing VA expressions, but could not always correctly tag all words that belong to the chunks. An additional striking finding is the fact that the models trained for the classification task (BERT-CLF) showed a lower performance than their binarized sequence tagging counterparts (BERT-SEQ-b), that is, the sequence tagging models trained on the same data.

We could show that, to some extent, the model is able to generalize in two directions. First, we analyzed the performance on the prediction of VA containing new syntactic phrases around the source. On the one hand, we found that the model could find new correct source phrases but on the other hand, the error rate is higher on such instances. A highly interesting outcome is that we found new variants of source phrases and that the model is able to identify such new phrases which can help future studies to expand the analyses of VA. Second, we analyzed whether the model is able to predict sources that are not limited to human entities (as is the case for the training data). We could show that the model indeed found new entity types, especially locations, fictional characters, companies, and brands. Compared to the source phrase analysis, all scores were much higher which indicates that this is an easier task for the model. To sum up, we conclude that our model is able to predict unseen VA types which can be used to expand the data and analyze the phenomenon further. As the evaluation of this study is sample-based, we have to be careful with the results. Still, both studies show promising results. The aVA dataset from Section 3.2 that emerged from manual annotations on the word-level is another contribution. The dataset is annotated and consists of 3,066 sentences that include VA expressions and 2,929 that do not include any. All chunks of the phenomenon are tagged, thus, this dataset can help to tackle further open challenges.

Since the phenomenon has not been studied deeply on an automated level, there are open challenges left, for example the extraction from non-English texts. The results of our work also provide new questions for future research, for instance, finding the full names of the targets to identify the corresponding entities, or identifying the characteristics of the source that the author wants to transfer to the target. A further goal is the automated generation of VA which depends on the transferred characteristics and is a future work we are going to explore. Different approaches to detect VA, for example, using a deeper semantic understanding, are a possible future project as well, although it is not as trivial as one could think. We already conducted preliminary experiments, for example, using different embeddings to analyze the semantic distance between VA chunks. The distance between source and modifier should be larger than the distance between target and modifier, as the modifier establishes the transfer to a context different from the source's. But there are too many exceptions, like semantically neutral modifiers, (for example, time-related like “his time” or “her era”), or modifiers that are a specification of the source's expertise and thus, still close to the source, as in “The Tiger Woods of mini golf”.

In conclusion, we proposed an end-to-end method to extract Vossian Antonomasia on the word-level that is able to tag all parts of the phenomenon and is robust against real-world data.

## Data Availability Statement

The datasets of this study can be found at https://vossanto.weltliteratur.net/ and in the Supplementary Material.

## Author Contributions

FF contributed mainly in the annotation processes and helped to write the section 1. MS conducted the whole analysis and wrote the main part of the paper. RJ and MS designed the studies together. RJ helped annotating data and wrote the paper. All authors contributed to manuscript revision, read, and approved the submitted version.

## Conflict of Interest

The authors declare that the research was conducted in the absence of any commercial or financial relationships that could be construed as a potential conflict of interest.

## Publisher's Note

All claims expressed in this article are solely those of the authors and do not necessarily represent those of their affiliated organizations, or those of the publisher, the editors and the reviewers. Any product that may be evaluated in this article, or claim that may be made by its manufacturer, is not guaranteed or endorsed by the publisher.

## Footnotes

^1^The title is another example. Like Rodney Dangerfield (source), a former comedian, VA (target) is often used in funny and provocative ways. Dangerfield's famous quote “I don't get no respect” fits very well to VA as in our opinion the phenomenon gets too little attention and respect in the field of stylistic devices (modifier).

^2^To avoid a long reference list, all examples taken from the New York Times corpus (Sandhaus, 2008) are cited using the pattern “NYT year/month/day/article-id”.

^3^See https://fivethirtyeight.com/features/hillary-clinton-is-the-george-w-bush-of-2016/.

^4^Instructed by a domain expert with 10 years of experience in the research of VAs.

## Abbreviations

VA: Vossian Antonomasia
CLF: classification
SEQ: sequence tagging
TRG: target
SRC: source
MOD: modifier
aVA dataset: annotated VA dataset
eVA dataset: enriched VA dataset
SIG dataset: Signal Media sample dataset
NYT: New York Times
{eVA, aVA}-TR: {eVA, aVA} training dataset (e.g., eVA-TR-50)
{eVA, aVA}-TE: {eVA, aVA} test dataset (e.g., eVA-TE-50)
LSTM: long short-term memory
CRF: conditional random field
BLSTM-ATT: bidirectional LSTM model with an attention mechanism on top (classification model)
BLSTM-CRF: bidirectional LSTM model with a CRF on top (sequence tagging model)
BERT-CLF: fine-tuned BERT classification model
BERT-SEQ: fine-tuned BERT sequence tagging model
BERT-SEQ-b: binarized fine-tuned BERT sequence tagging model.
